# The PTH1R variant H442D co-segregating with PFE localizes to cell membranes but fails to elicit adequate PTH-dependent cAMP levels

**DOI:** 10.1186/s40510-026-00653-y

**Published:** 2026-11-06

**Authors:** Matthias Weider, Tomas Taraba, Gabriele Rodrian, Michael Hofmeister, Lina Gölz

**Affiliations:** 1 Zahnklinik 3 – Kieferorthopädie, Universitätsklinikum Erlangen, Erlangen, Germany; 2 Lehrstuhl für Zahn-, Mund- und Kieferheilkunde, insbesondere Kieferorthopädie, Friedrich-Alexander-Universität Erlangen-Nürnberg, Erlangen, Germany

**Keywords:** PTH/PTHrP receptor type I, Primary failure of eruption, cAMP signalling, Orthodontics, Ankylosis

## Abstract

**Background:**

Primary failure of eruption (PFE) is a rare disorder characterized by failure of teeth to erupt, which leads to severe lateral open bite. It is often caused by heterozygous mutations in the parathyroid hormone receptor (*PTH1R*) gene. Although a variety of *PTH1R* mutants has been shown to be associated with PFE, molecular disease-causing mechanism are rarely identified.

**Findings:**

We here describe a co-segregation study and the cell biological characterisation of the PTH1R variant H442D that hitherto has been classified as of uncertain clinical significance. In a cohort study, we show that the H442D variant segregates with PFE. Moreover, while it displays correct localization to cell membranes, it evokes only modest levels of the downstream second messenger cAMP upon stimulation with PTH.

**Conclusions:**

Our study provides a molecular mechanism for the co-segregation of the PTH1R variant H442D with PFE. The decreased PTH-dependent cAMP signal may be causative for the observed PFE phenotype in subjects carrying this heterozygous mutation.

**Supplementary Information:**

The online version contains supplementary material available at https://doi.org/10.1186/s40510-026-00653-y.

## Introduction

Primary failure of eruption (PFE) is a rare disorder characterized by failure of teeth to erupt to the occlusal plane. First described in 1981 [1], PFE affects mainly posterior teeth and leads to severe lateral posterior open bite [2]. A distinction is made between PFE type I (with a progressive open bite from anterior to posterior) and type II (with distal teeth presenting greater – although inadequate eruption than the most mesial tooth) [2]. The disorder is predominantly associated with Angle class III malocclusions [3]. The treatment of PFE is complex and requires a multidisciplinary approach including prosthetics, maxillofacial surgery and orthodontics. Satisfying treatment results are hard to achieve [3]. One major problem during orthodontic treatment is ankylosis [1]. Thus, a detailed evaluation of the severity of each patient’s symptoms is necessary to select the best treatment option, with genetic diagnosis highly recommended [2, 4–6].

Heterozygous mutations in the parathyroid hormone receptor (*PTH1R*) gene have been demonstrated to cause PFE [4–10]. The mutations described so far lead to amino acid changes, premature stop codons or errors in mRNA splicing. PTH1R is activated by parathyroid hormone (PTH) and PTH-related peptide (PTHrP). Via PTH1R, PTH controls Ca^++^ homeostasis, while PTHrP regulates development of various tissues [11]. Both hormones mediate anabolic as well as catabolic bone remodelling processes via PTH1R. PTH1R is a classical seven transmembrane G-protein coupled receptor; downstream signalling pathways are the cyclic adenosine monophosphate (cAMP) – protein kinase A (PKA) pathway and inositol phospholipid – protein kinase C pathway [11].

Functional characterization of pathogenic variants is scarce [12–15] and the underlying disease-causing mechanisms remain unknown for the majority of variants. As knowledge of the phenotypical severity of the different *PTH1R* variants is important for treatment, we set out to gain further evidence for co-segregation of PFE with a variant with unclear clinical significance that had been identified in one of our patients. Moreover, we strove to gain insights into the molecular disease-causing mechanism of this variant.

## Results and discussion

A patient from our clinic suffering from PFE (Tables 1 and 2) was diagnosed in a Human geneticists PFE panel. This report identified a heterozygous base exchange from cytosine to guanine at position 1324 (c.1324C > G) of the *PTH1R* coding sequence (CDS). No other PFE risk gene included in the panel was mutated. The mutation c.1324C > G which leads to the amino acid exchange of histidine 442 to aspartate (H442D) was classified as variant of uncertain clinical significance (ACMG3) according to published literature [8]. We therefore set out to (1) gain further proof for the co-segregation of this variant with PFE and (2) provide a cell biological characterization of this variant.

**Table 1:**
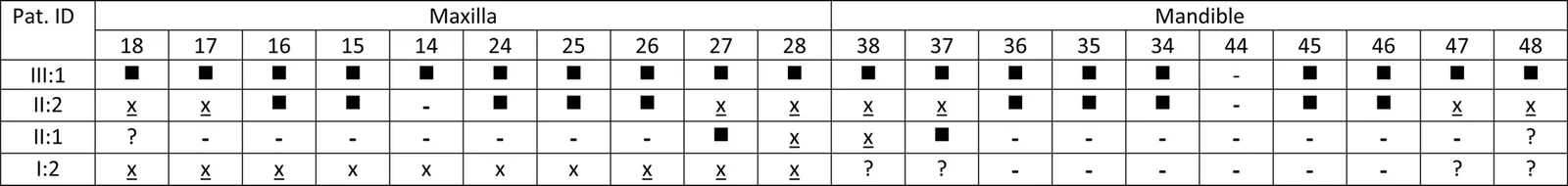
Characterization of family members with *PTH1R* mutations. ■  = affected teeth; x = extracted teeth very likely to have been affected; x = extracted teeth very likely not to have been affected; - = unaffected teeth

**Table 2:**
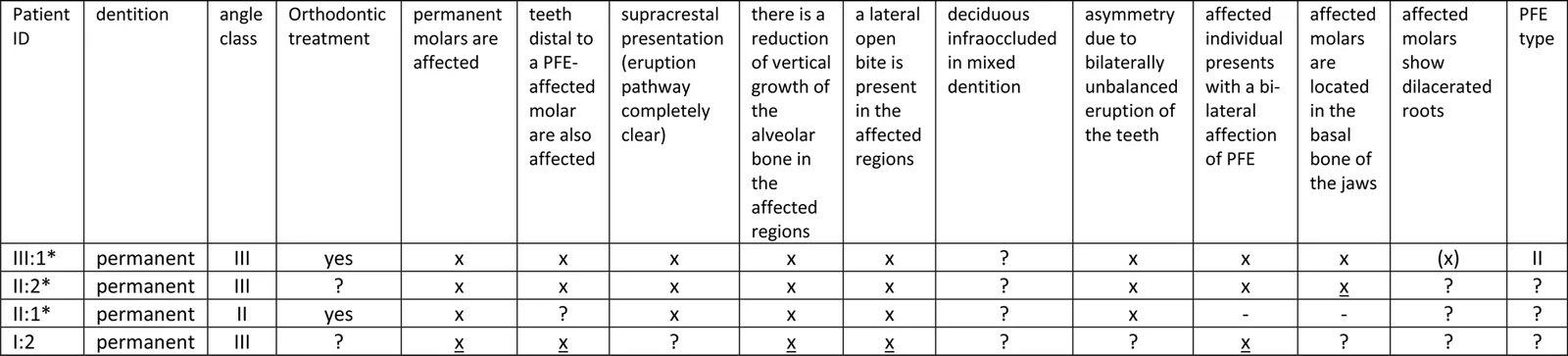
Clinical signs in family members with *PTH1R* mutations according to Grippaudo et al. [5]. *patients that show at least 5 of the PFE hallmark signs; x = present; - = not present; (x) = not all (only teeth 37 and 46); ? = no data available and patients did not remember; x = likely (based on intraoral findings and clinical documentations)

**Fig. 1:**
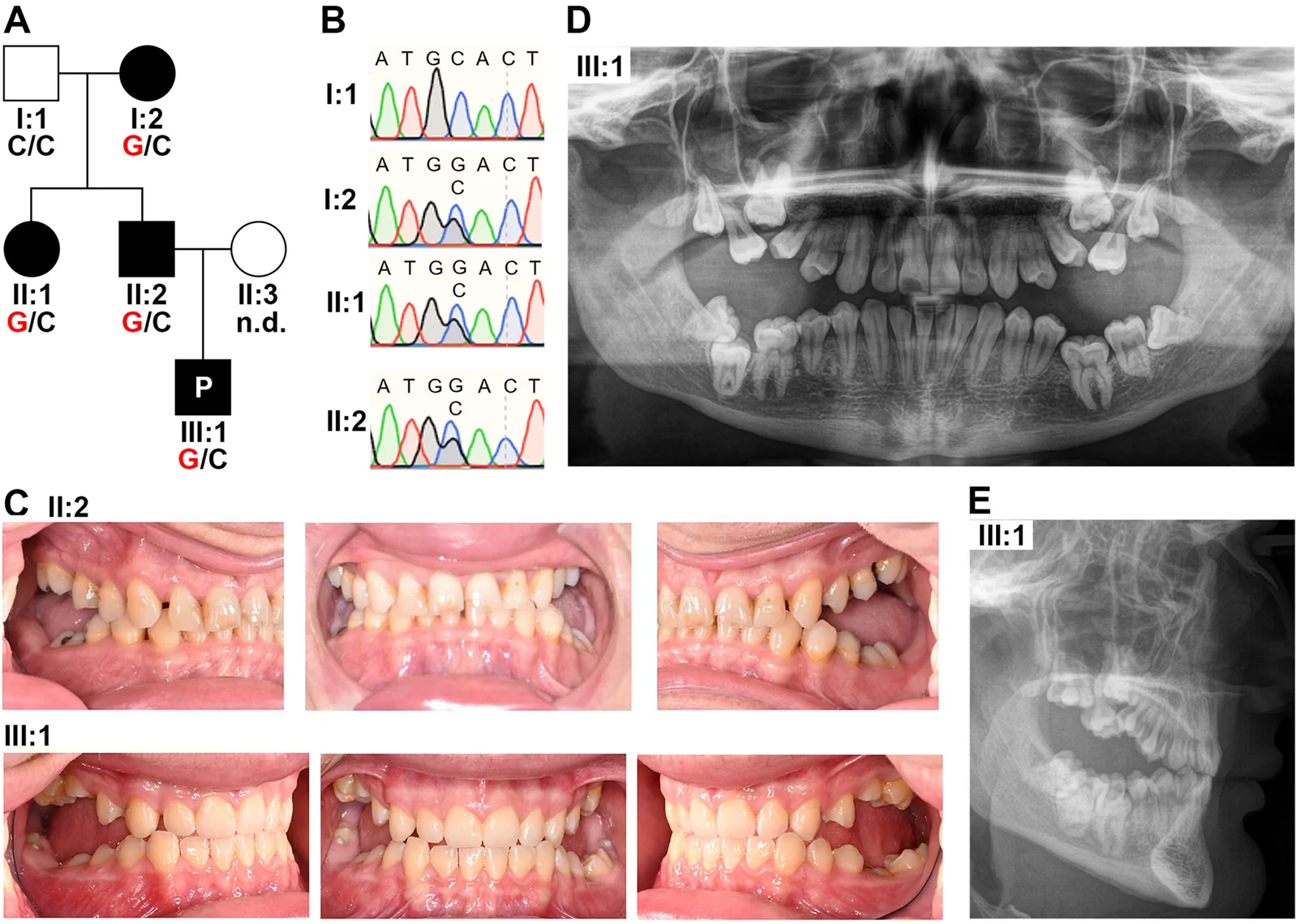
The PTH1R variant H442D co-segregates with PFE. (**A**) Schematic pedigree depicting the segregation of clinical phenotype and PTH1R variant H442D. The relationship of subjects to index patient (**P**) and their allelic composition at bp 1324 of *PTH1R* CDS is shown. Circles and squares represent female or male subjects, respectively. Filled symbols represent PFE cases while open symbols represent healthy subjects. n.d. = not determined. (**B**) Partial subjects’ DNA sequences around bp 1324 of *PTH1R* CDS as identified by Sanger sequencing. (**C-E**) Intraoral photographs of patients II:2 and III:1 (**C**), orthopantomogram of patient III:1 (**D**) and lateral cephalogram of patient III:1 (**E**) show eruption failure of posterior teeth and deficits in alveolar bone growth, resulting in bilateral open bite

To characterize the co-segregation of variant H442D with clinical PFE symptoms, we performed sequencing of genomic DNA around base pair 1324 of *PTH1R* CDS of the index patient’s relatives. We generated a pedigree across three generations including three relatives of the index patient that had been diagnosed for PFE (Tables 1 and 2) as well as one healthy relative as control (Fig. 1A–E; Table 1). As expected, each PFE patient displayed heterozygosity for bp 1324 while the healthy subject carried the homozygous wild type (WT) allele. We conclude that the *PTH1R* mutation c1324C > G coding for variant H442D co-segregates with PFE.

**Fig. 2:**
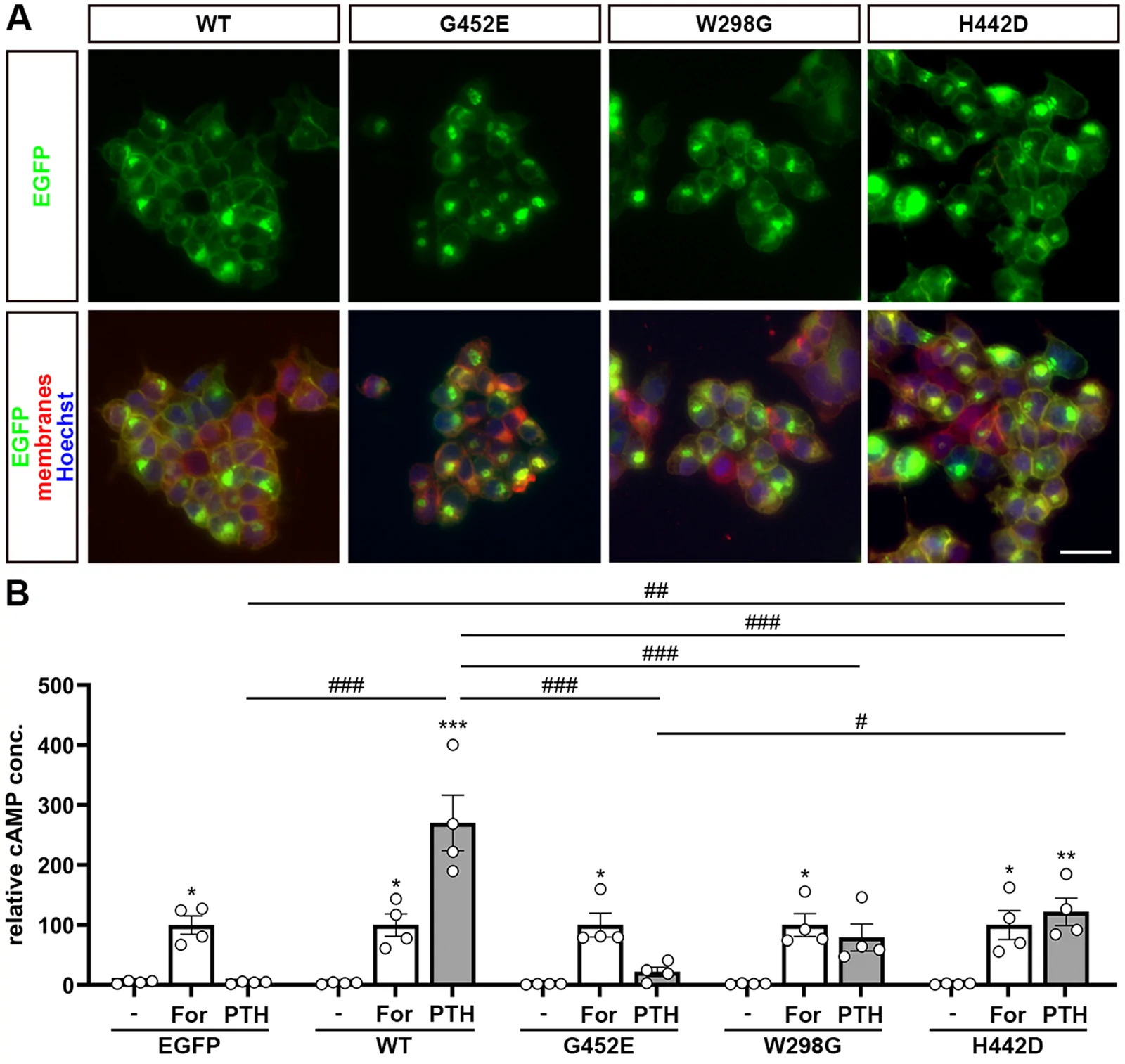
PTH1R variant H442D localizes to cell membranes but confers only modest cAMP responses to PTH. (**A**) Representative images of HEK293T cells transfected with expression plasmids for C-terminal EGFP-fusions of PTH1R variants WT, G452E, W298G and H442D (green). Cellular membranes were counterstained with CellMask orange (red), nuclei were counterstained with NucBlue Live containing Hoechst 33​342 (blue). (**B**) Relative cAMP levels of HEK293T cells that had been transfected with expression vectors for EGFP (negative control) or the PTH1R variants WT, G452E, W298G or H442D and that were stimulated with Forskolin (for), PTH or not stimulated (-) as determined by ELISA. Graph bars represent arithmetic means ± SEM (*N* = 4), means of Forskolin-stimulated samples of each variant were arbitrarily set to 100%. Asterisks (*) depict statistically significant differences to non-stimulated control with the same PTH1R variant, hashtags (#) depict statistically significant differences between PTH-stimulated samples. Statistical significance was determined by one-way ANOVA with Tukey’s post correction. *0.01 ≤ *p* < 0.05, **0.001 ≤ *p* < 0.01, ***p < 0.001; ^#^0.01 ≤ *p* < 0.05, ^##^0.001 ≤ *p* < 0.01, ^###^*p* < 0.001. Scale bar: 50 µm

In order to gain insight into the disease-causing mechanism of this mutation, we next set out to identify the subcellular localization of this PTH1R variant. To this aim, we transfected HEK293T cells with expression plasmids for C-terminal EGFP fusions to WT PTH1R, the H442D variant and the G452E variant as control, as this variant had been shown to localize mostly to internal cellular structures [15]. Moreover, we included the missense variant W298G that also had been shown to co-segregate with PFE [16] as an additional control. Cellular membranes were counterstained with a pan-membrane stain. As expected, WT PTH1R localized to the cell membrane and the G452E variant was stuck in internal cellular structures (Fig. 2A). Interestingly, the H442D variant as well as the W298G variant localized to the cell membrane.

As our index patient’s variant showed correct intracellular localization in our assay, we asked whether reduced signal relay capability might be a possible disease-causing mechanism of the H442D variant. We therefore analyzed if PTH-dependent cAMP concentrations were affected, thus directly tracking an intracellular second messenger involved in physiologic PTH1R signalling during tooth eruption [14]. To this aim, HEK293T cells were transfected with expression plasmids encoding PTH1R variants or EGFP as negative control. We stimulated these cells with PTH and measured intracellular cAMP concentrations. Forskolin served as control for PTH1R-independent stimulation of adenylate cyclase. WT PTH1R mediated a drastic PTH-dependent increase of cAMP levels (Fig. 2B). The variant G452E stimulated only a very small increase over the unstimulated situation that did not reach significance compared to the unstimulated situation or the PTH-stimulated negative control. This finding is in agreement to its localization in internal cellular structures and with the literature where it had been shown to elicit only very weak phosphorylation of vasodilator-stimulated phosphoprotein at serine 157 [15]. Intriguingly, variant H442D evoked a significant, but drastically reduced PTH1-dependent increase of cAMP levels compared WT PTH1R (1.22±0.23-fold of Forskolin in H442D compared to 2.70 ± 0.46-fold in WT). Consistent with the similar intracellular localization, we detected comparable results for the W298G variant that showed no significant difference to the H442D variant (0.79±0.23-fold of Forskolin). As cAMP-PKA-signalling is one major pathway through which PTH stimulates both osteogenesis and osteoclastogenesis [17, 18], it is plausible that the reduced cAMP signalling capacity of the H442D mutant leads to the PFE phenotype observed in our patient and his affected relatives. Determination of cAMP levels was also used to characterize another PFE-associated *PTH1R* variant in a recently published work [19]. A limitation of our study is that the results were gained by overexpression assays in a heterologous system, which does not fully establish the in vivo disease mechanism in dental/periodontal tissues. Nevertheless, the very similar results in both assays gained with the already identified W298G variant [16] to the H442D variant underscore the importance of our findings. Future research might address the question whether differing degrees of residual cAMP signalling across PTH1R variants could contribute to variable clinical severity. Of note, both variants with residual cAMP responses nevertheless can evoke massive clinical phenotypes (Table 1) [16].

Histidine 442 resides in the extracellular portion of helix VII of PTH1R, which has a critical role for binding of PTH [20]. Of note, it resides directly next to methionine 441 that fulfils an important function in PTH binding [20]. It is therefore highly conceivable that substitution of histidine 442 to aspartate in the H442D variant impacts binding of PTH and PTHrP to the receptor, leading to reduced cAMP levels and ultimately evoking PFE.

Collectively, our study on the PTH1R variant H442D (1) confirmed its co-segregation with PFE and (2) for the first time provides a cell biological mechanism for this co-segregation and thus offers important insights into the molecular mechanism of PFE.

## Materials and methods

### Genealogic analyses

The patient’s c.1324C > G mutation was identified in a Human geneticist’s report. No other mutations in PFE risk genes included in the panel (i.e. *CEP152, CLDN1, EDA, EDAR, EDARADD, MSX1, PAX9, POLR3A, POLR3B, PORCN, PTH1R, SATB2, TP63, WNT10A*) were observed. For sequence analysis of the patient’s relatives that had been diagnosed as PFE patients or as healthy, genomic DNA was extracted from whole blood with QIAamp DNA blood mini kit (Qiagen) and amplified by PCR around bp 1324 of CDS with primers listed in Supplementary Table 1. PCR products were separated on agarose gels, the 330 bp bands were excised, purified via QIAquick PCR & Gel Cleanup Kit (Qiagen) and analyzed by Sanger sequencing. Clinical PFE features were confirmed by a specialist in Orthodontics from our department.

### Cloning procedures

Expression plasmid containing WT human *PTH1R* CDS was purchased from OriGene. Mutants coding for variants H442D, G452E and W298G were generated by site-directed mutagenesis with Q5 kit (NEB) with primers listed in Supplementary Table 1. Correct base exchanges and preservation of all other CDS bases were confirmed by Sanger sequencing. For C-terminal EGFP-fusions, CDS’s encoding PTH1R variants were cloned into pEGFP-N2 (Clontech) with NheI and BamHI by standard cloning procedures.

### Analysis of intracellular PTH1R-localization and cAMP concentrations

HEK293T cells were grown on Gelatine-coated plasticware in DMEM (Gibco) containing 10% FCS, transfected with Lipofectamine 2000 (Invitrogen) and further analyzed 24 h post-transfection. For subcellular localization studies, cells were transfected with EGFP-fusions of PTH1R variants. Cellular membranes were labelled with pan-membrane stain CellMask Orange (Thermo Fisher) and nuclei were counterstained with NucBlue Live (Thermo Fisher). Fluorescence images were taken on a Zeiss Axio Vert.A1 microscope. For the determination of intracellular cAMP levels, cells were transfected with expression plasmids for PTH1R variants or EGFP as negative control. Stimulation was performed after 2 h starvation in serum-free medium with 10 nM PTH (Tocris), 5 µM Forskolin (Abcam) or no additive (negative control) for 15 min in serum-free medium containing 1 mM of the phospho-diesterase inhibitor IBMX (Sigma-Aldrich). After lysis with 0.1 M HCl, cAMP values were determined with the Cyclic AMP Select ELISA kit (Cayman Chemicals) according to the manufacturer’s instructions and measured on a Synergy H1 multimode microplate reader (BioTek). Measured cAMP levels were set in relation to total protein concentrations as determined by DC assay to calculate cAMP concentrations. Mean values of cAMP concentrations of Forskolin-stimulated samples for each variant were arbitrarily set to 100% (*N* = 4). Statistical analysis was performed with one-way ANOVA with Tukey’s post correction over all samples.

## Electronic supplementary material

Below is the link to the electronic supplementary material.

Supplementary material 1: PCR primers used in this study

## Data Availability

All data generated or analysed during this study are included in this published manuscript.

## Abbreviations

cAMP: Cyclic adenosine monophosphate
CDS: Coding sequence
PFE: Primary failure of eruption
PKA: Protein kinase A
PTH: Parathyroid hormone
PTH1R: Parathyroid hormone receptor 1
PTHrP: PTH-related peptide
WT: Wild type
